# The Integrative Method “Suture Dragging and Simplified Vacuum Assisted Therapy” for Complex Pilonidal Sinus Disease

**DOI:** 10.1155/2014/425497

**Published:** 2014-03-04

**Authors:** Chen Wang, YiBo Yao, YongQing Cao

**Affiliations:** Department of Anorectal Surgery, Longhua Hospital, Shanghai University of Traditional Chinese Medicine, No. 725 South Wanping Road, Shanghai 200032, China

## Abstract

Complex and recurrent pilonidal sinuses are best treated with surgery. Different surgical modalities as complete excision of the pilonidal sinus leave the wound open or procedures like closing the wound with or without reconstructive flap are widely used. The open procedure is radical but may cause broad excision and prolonged morbidity, while risk of infection and rate of recurrence are higher in the closed techniques. Traditional Chinese surgical treatments are less invasive and more effective; they have been used to treat sinus and fistula disease successfully. In this case report, we have described a male adolescent with complex pilonidal sinus, who received traditional Chinese surgical treatment combined with modern wound healing technique. He recovered completely with short hospitalization, good tolerance, less pain, and scarring. Therefore, we recommend using this integrative method to treat complex pilonidal sinus disease.

## 1. Introduction

Pilonidal disease was first described by Hodges in 1880. It is diagnosed by finding a characteristic epithelial track situated in the skin of the natal cleft, a short distance behind the anus and generally containing hair [1]. The onset of pilonidal sinus (PNS) usually begins between the age of 15 and 40. The incidence in males is nearly ten times that in females [2]. The etiology is unclear; hormones, hair, friction, and infection may be the majority of pathological origins of PNS. Male sex, adolescence of youth, familial disposition, local trauma, and overweight seem to be associated with the development of PNS [3]. Acute PNS needs to be drained, may include debris removal and wound packing. Complex and recurrent PNS are best treated with surgery. Clinically, patients with complex PNS are more common. Low recurrence rate, minimal operation time, convenience, short time off work, and cost are important considerations [4].

The number and variety of published techniques are testament to the complexity of treating PNS and the fact that no single procedure is superior in all respects. The surgical management of the complex and recurrent PNS is controversial. The majority of procedures can be classified into first, excision and healing by second intention, and second, excision with primary closure or reconstructive flap. The advantage of the first procedure is low recurrence, but the downside is a prolonged healing time (8–10 weeks) [5]. Closure of the wound after excision is more cosmetically acceptable for patients and is associated with a shorter healing time but coupled with high risk of postoperative infection [6]. Meanwhile, the reconstructive flap procedure is more technically demanding and is probably best performed by an experienced colorectal surgeon [7]. Achieving cure in a timely manner and minimizing the recurrence rate with good tolerance would be the objectives of surgical treatments for PNS.

## 2. Case Presentation

**Figure 1:**
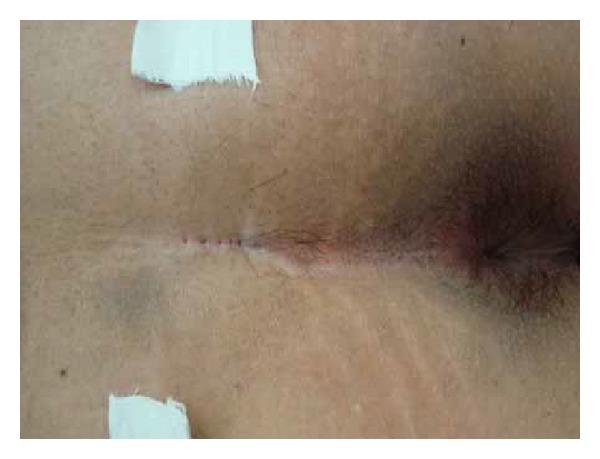
The complex pilonidal sinus with several openings and extended abscess.

A 16-year-old boy with discharge and pain behind the anus was admitted to the hospital. He had sepsis at the same place for approximately 1 year and underwent spontaneously drainaged 3 month ago. Several pilonidal sinus openings were noted in the midline, overlying the coccyx. A lump of tenderness was found left lateral to the openings (Figure 1). Ultrasound revealed a 3 cm length sinus track beneath the openings which extend to a 2.2 cm × 2.0 cm sepsis left laterally. There was no internal opening to the anal canal.

**Figure 2:**
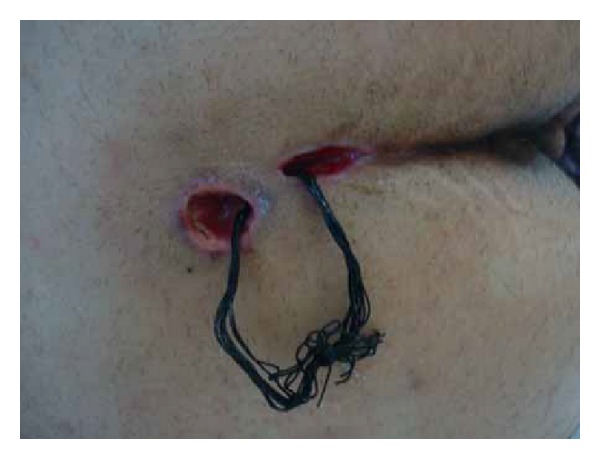
The sutures in the sinus tract cavity.

**Figure 3:**
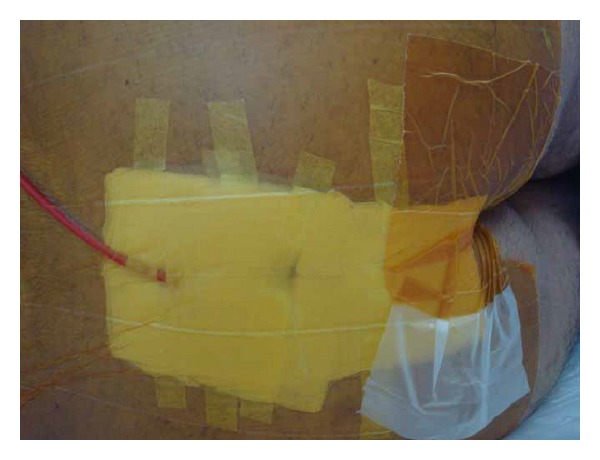
The wound with simple vacuum assisted dressing in place.

**Figure 4:**
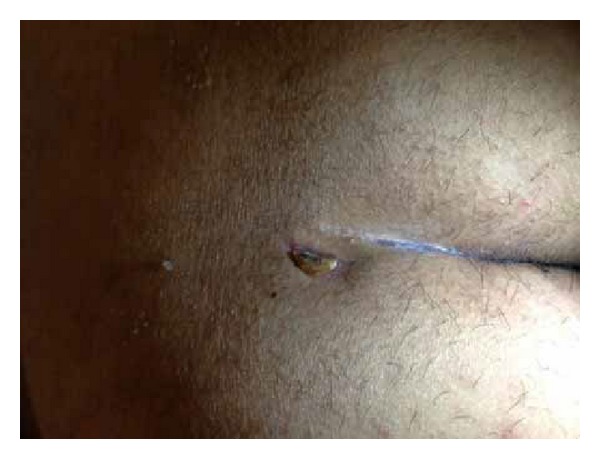
Three weeks after the operation, the reepithelialization was complete.

The patient was placed in prone position under spinal anesthesia. One dose of metronidazole was used intravenously during operation. A blunt probe was gently placed through the sinus openings at the midline. The tip of the probe extends to the abscess; tufts of hair were found confirming that this represented pilonidal disease. Anoscopy was then performed and the anal canal was inspected in its entirety. The anal canal was normal; there was no evidence of disease. A 3 cm ellipse incision was made by a blade to remove the skin, subcutaneous tissues, and the pilonidal sinus tract *en bloc*. A counter incision was made at the extension of sepsis to drain. There was little pus and infected granulation tissue presented in the sinus track, which was submitted to pathological evaluation. The wound was then irrigated with normal saline and hemostasis was obtained. Ten 2-0 silk sutures were put through the 2 incisions and secured loosely to themselves (Figure 2). A clean, dry, sterile dressing was placed over the top. Patient was given intravenous metronidazole daily for three days postoperatively. Dressings were changed by doctor twice per day at 8 am and 4 pm, respectively. The suture dragging process started from the first day after surgery during dressing change. After irrigation with normal saline, part of the silk sutures which were inside the sinus tract was dragged out and cleaned then turned back into the cavity. All sutures were removed at day 8 postoperatively when the wound became fresh with minimal drainage. Then a 16# straight red rubber catheter with hospital vacuum system was used to assist the wound healing. The top of the catheter was cut into oblique shape and fixed by tape at the edge of the abscess wound. A 5 cm × 5 cm sterile cotton gauze was folded and put at both sides of the wound. Then 3 pieces of sterile gauze were put on the surface of the sinus cavity. A 15 cm × 15 cm antimicrobial incise drape (Ioban 2, 3 M Health Care, MN, USA) covered catheter and gauzes for seal (Figure 3). The catheter was connected to a hospital vacuum system applied with continuous negative pressure (−30 kPa) to close the wounds. The dressing and catheter were changed and wounds were cleaned every 2 days. The 14# and 12# catheters were used in sequence to correspond to the size of the wound cavity. The vacuum assisted therapy was stopped untill the wound cavity contract noticably 1 week later and patient was discharged. Complete wound reepithelialization was obtained at 22 days postoperatively (Figure 4). Repeated ultrasound confirmed no track and sepsis at the natal cleft. The patient recovered well and remained asymptomatic for 12 months.

## 3. Discussion

The incidence of PNS in China is low, but misdiagnosis rate and recurrence rate are high. The recurrence rate of PNS is from 5 to 40 percent, with the highest rates in complex sinus tracts [8]. The ideal treatment would at least meet the following criteria: ease of performance, short duration of wound healing, low recurrence rate, minimal pain and wound care, fast return to normal activity, and cost effectiveness [9]. The treatment options for complex pilonidal sinus include excision with healing by secondary intention or plastic surgical procedures to obliterate the defect. However, prolonged morbidity and high recurrence rate are the main defects of these treatments. Traditional Chinese surgical therapies, such as suture dragging and pad compression, had been used for more than 40 years to deal with the sinus and fistula disease. These traditional methods preserve the integrity of musculature involved by sinus and fistulous tract and maintain good function with less postoperative pain and shorter healing time [10, 11].

In this case, the traditional Chinese surgical treatment “suture-dragging therapy” was adapted to treat the complex PNS. Instead of broad excision and muscle flap, suture dragging just needed appropriate incision and counter drainage, which were minimal invasive. By dragging the sutures during dressing change, pus and necrotic tissue could be drained completely. Though suture-dragging therapy was less invasive, how to speed sacrum wound cavity healing still poses a considerable challenge to colorectal surgeons. Literature showed that both positive and negative pressures have been proved to accelerate the wound healing by increasing local blood flow and the rate of granulation tissue formation [12–15]. Recently, vacuum assisted closure technique like VAC has been used by plastic surgeons to facilitate the healing of chronic or complicated wounds [16]. The VAC device consists of a foam pad in the internal shape of the wound which is inserted with plastic fenestrated tube applied to the center. In this case, inserting foam pad to the primary wound cavity was not infeasible. We used different sizes of the red rubber catheter to correspond to the diminishing wound cavity, connected to the hospital vacuum system, and sealed with an antimicrobial incise drape. This simplified vacuum assisted method is effective, economical, and easy to perform.

Suture-dragging and simple vacuum assisted therapy are integrative methods of complex pilonidal sinus disease. They shorten the duration of wound healing and provide cosmetically acceptable result with no need for further surgical treatment.
